# Ultrasound-guided quadratus lumborum block for perioperative analgesia in robot-assisted partial nephrectomy: a randomized controlled trial

**DOI:** 10.1186/s13063-021-05815-3

**Published:** 2021-11-24

**Authors:** Renchun Lai, Quehua Luo, Jielan Lai, Xiaoyun Lu, Mei Xu

**Affiliations:** 1 Department of Anesthesiology, Sun Yat-sen University Cancer Center, State Key Laboratory of Oncology in South China, Collaborative Innovation Center for Cancer Medicine, Guangzhou, 510060 People’s Republic of China; 2 Department of Anesthesiology, Guangdong Provincial People’s Hospital, Guangdong Academy of Medical Sciences, Guangzhou, Guangdong People’s Republic of China

## Abstract

**Background:**

Recently, several case reports and limited randomized studies have shown that quadratus lumborum blocks (QLB) are effective in providing pain relief after intra-abdominal and retroperitoneal operations. Additionally, robot-assisted partial nephrectomy (RAPN) has been proposed as a promising operative treatment for renal carcinoma because it enables early recovery and ambulation. Therefore, we aimed to evaluate the analgesic and opioid-sparing effects of a single-injection QLB in patients undergoing RAPN to determine its role in an early recovery program.

**Methods:**

Fifty-six patients undergoing elective RAPN under general anesthesia were randomized to two equally sized groups. Patients were randomly allocated to receive either a unilateral QLB (*n* = 28) with 0.375% bupivacaine 0.5 mL/kg (QLB group) or a conventional scheme (*n* = 28) (control group). The QLB technique, termed QLB2, was performed as first described by Blanco. The primary outcome was visual analog scale (VAS) scores with movement at 6 h postoperatively. The secondary endpoints were morphine consumption at different time periods after surgery, morphine-related side effects, and assessment of postoperative rehabilitation.

**Results:**

Both the VAS pain scores and cumulative opioid consumption were significantly lower in the QLB group at 6 h after surgery as compared with results in the control group (all *P* < 0.05). There were significant differences in pain scores at all time points except at 4 h with movement and 48 h at rest. However, at 12–24 h no significant differences between the two groups were observed in cumulative opioid consumption or in the duration of PACU and hospital stays. The patient recovery scores were significantly higher in the QLB group.

**Conclusions:**

Single-injection pre-emptive QLB applied to RAPN was effective and provided satisfactory analgesia and opioid-sparing effects in combination with typical patient-controlled analgesia. In addition, it may provide an effective technique for early recovery in the perioperative period for RAPN.

## Background

The quadratus lumborum block (QLB) was first described by Blanco in 2007, and later was subdivided into three basic approaches known as QLB1, 2, and 3, as well as a modified approach called intramuscular QLB, each based on needle tip position and the spread of local anesthetic (LA) [1]. Recently, numerous case reports and randomized trials have shown that QLB may be effective in relieving postoperative pain and providing an opioid-sparing effect after various surgical procedures [2–10].

Although the analgesic effects are theoretically thought to be comparable among the four types of QLB, in fact each approach results in a different range of sensory blockades due to how the spread of LA varies for each method. QLB2, also termed the posterior approach, has been reported to be effective for both somatic and visceral pain due to the spread of LA to the paravertebral space, and it is believed to achieve a sensory blockade of the ipsilateral abdominal wall (lateral and lower) [11, 12]. Furthermore, the QLB2 technique calls for an injection of LA into the fascial plane between the quadratus lumborum and the latissimus dorsi muscles. The superficial point of this LA injection allows real-time ultrasound guidance, making this method safer and more user-friendly. Thus, analyzing its clinical efficacy under different clinical procedures to determine its potential applications is critical.

The past decade has seen the rise of robot-assisted partial nephrectomy (RAPN) used for the treatment of renal carcinoma, as it has the advantages of shortening warm ischemia time and providing a better solution for difficult-to-access renal tumors [13–16]. It acts as a minimally invasive technique and is now an important part of an early recovery program. Several case reports have shown that QLB was effective in providing pain relief after unilateral abdominal surgeries (e.g., pyeloplasty and radical nephrectomy [17, 18]). However, a literature search did not find any randomized, controlled trials that evaluated the effect of QLB after RAPN. In this randomized, controlled, double-blinded study, we investigated our hypothesis using the QLB2 technique in combination with a typical postoperative analgesic scheme to evaluate the clinical efficacy on perioperative analgesia in RAPN.

## Methods

### Design and patients

**Fig. 1:**
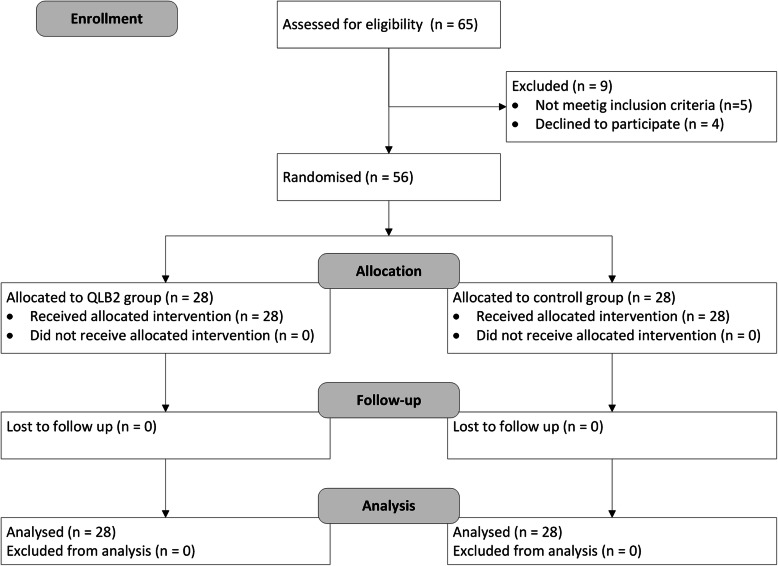
Patient enrollment and randomization

Ethical approval for this study was provided by the Research Ethics Committee of Sun Yat-sen University Cancer Center (Chairperson: Prof. Wangqing Peng) on 5th June 2018. The study was registered at a clinical trials registry (www.chictr.org.cn, registered number: ChiCTR1800016790), and was conducted at the Sun Yat-sen University Cancer Center from June 2018 to November 2018. This study was designed and conducted on the basis of the Consolidated Standards of Reporting Trials (CONSORT) guidelines. Patients meeting the American Society of Anesthesiologists (ASA) physical status I to III, aged 18 to 75 years old, scheduled for elective RAPN, and receiving general anesthesia were enrolled in the study (Fig. 1). Exclusion criteria included: patient’s refusal, mental illness, history of alcohol or analgesic dependence, allergy to local anesthetics, severe hepatic dysfunction, coagulopathy, chronic pain, infection at the needle insertion site, and body mass index (BMI) more than 30 kg/m^2^ or less than 15 kg/m^2^. Written informed consent was obtained from all patients for this trial. The patients were randomly allocated by a computer-generated randomization schedule into one of the two groups (QLB group and control group). The QLB group received a unilateral QLB after anesthesia induction, and no nerve block was performed in the control group.

A sealed opaque envelope containing a card with the computer-generated allocation number (1 = QLB group, 2 = control group) was opened by an anesthesia assistant with no involvement in the study. Ultrasound-guided QLB was carried out by attending anesthesiologists who had extensive experience with this technique (over 60 attempts). Other anesthesiologists who were blinded to the patient group assignments were responsible for anesthesia management and pain management in postoperative care based on the specified scheme of anesthesia and analgesia. The research assistants who were engaged in the study performed the 48-h postoperative follow-up and recorded the clinical variables.

### Anesthesia and analgesia protocol

All patients were seen two days before surgery and were instructed on how to use a patient-controlled analgesia (PCA) device and the use of an 11-point visual analog scale (VAS) for the assessment of postoperative pain: 0 = no pain, ranging to 10 = worst imaginable pain. Prophylactic analgesia was performed the night before the surgery for all patients with celecoxib 200 mg, except for those with contraindications.

Upon arrival in the preparation room, peripheral venous access (20-gauge) was established, and standard ASA monitors were applied. Additionally, each patient received routine continuous monitoring of bispectral index (BIS) and train-of-four (TOF) stimulation. General anesthesia was induced with a target-controlled infusion (TCI) of propofol 3–6 μg/mL and intravenous sufentanil 0.3 μg/kg, and tracheal intubation was facilitated with rocuronium 0.9 mg/kg. A total intravenous anesthesia with TCI propofol and remifentanil was used for maintenance of anesthesia in accordance with BIS 40–60. A neuromuscular blockade was ensured throughout the surgical procedure using rocuronium according to a TOF index for adequate muscle relaxation. After intubation, the lungs were ventilated at 8 mL/kg tidal volume with 0–5 mmHg positive end-expiratory pressure. The frequency was set to keep the end-tidal carbon dioxide within the range of 35–45 mmHg. An infusion of phenylephrine between 0.1–0.4 μg/kg/min was initiated to maintain blood pressure values within 20% of the baseline value, and bradycardia (heart rate < 50 beats/min) was treated with atropine 0.5 mg. Dexamethasone 8 mg combined with tropisetron 5 mg was used to treat nausea and vomiting. A single bolus of sufentanil 0.1 μg/kg and flurbiprofen 50 mg was administered at the start of skin closure. All patients were extubated prior to transport to the post-anesthesia care unit (PACU).

At the PACU, a dose of intravenous morphine 2 mg was administrated for rescue analgesia if the VAS pain score was > 3 and was continued every 15 min until the VAS score fell to ≤ 3. Patients were transferred to the surgical ward when they achieved a modified Aldrete score of ≥ 9 and a VAS score of ≤ 3. All patients routinely received a morphine PCA pump with no background infusion. The PCA pump was programmed to deliver 1 mL bolus doses of morphine (1 mg/mL) on demand with a minimum lock-out interval of 6 min, and a total morphine dose not to exceed 15 mg during any 4-h interval. All patients received regular flurbiprofen 50 mg at 8-h intervals and a dose of intravenous tramadol 50 mg was defined for breakthrough pain if the VAS score was ≥ 6, every h until the VAS was ≤ 3. Antiemetic treatment with intravenous tropisetron was available as needed.

### Ultrasound-guided QLB2 technique

All blocks were performed after induction of general anesthesia with the patient in the lateral decubitus position. After local sterilization with povidone iodine, a high-frequency (6–13 MHz) linear array ultrasound probe (Sonosite M-turbo, SonoSite, Inc., Bothell, WA, USA) covered with a sterile sheath was placed above the iliac crest and moved cranially until the three abdominal wall muscles were clearly identified. Then, it was slid medially until latissimus dorsi and quadratus lumborum muscles were confirmed within identical short-axis views. A 22-gauge, short-beveled stimulating needle (B. Braun Melsungen AG, Melsungen, Germany) was used for the single-injection block. The needle tip was inserted in-plane with an ultrasound beam targeting the posterior of the quadratus lumborum muscle. Accurate needle tip position was initially readjusted by injecting 1–2 mL of normal saline for hydrodissection image guidance, and 0.5 mL/kg of 0.375% bupivacaine was injected into the lumbar interfascial triangle behind the quadratus lumborum muscle under real-time ultrasound guidance.

### Measurements

All patients underwent RAPN using a Da-Vinci Si system with 4 to 6 port holes. Demographic, anesthesia- and operation-related parameters were recorded, including sex, age, BMI, height, weight, performance time, surgical duration, and consumption of intraoperative propofol and remifentanil. The primary outcome measure of this study was the VAS scores during movement (coughing or turning the body) at 6 h postoperatively. After surgery, patients were evaluated at 4, 6, 12, 24, and 48 h. The measured variables were VAS scores during rest and with movement at the predetermined intervals; morphine titration consumption in the PACU and cumulative morphine PCA consumption; length of PACU stay; morphine-related side effects including nausea, vomiting, sedation (Ramsay scale > 2), dizziness, hypoxemia (pulse oxygen saturation < 95%), and pruritus; and additionally, hospital stay duration and quality of recovery (measured using a 15-item quality of recovery score (QoR-15)) were also documented.

### Sample size calculation and statistical analysis

The sample size was calculated based on data from our pilot study (n = 10). We observed that the VAS score (during movement) at 6 h postoperatively was 4 (±2.0). We considered a 50% reduction in the VAS scores to be statistically significant, and therefore 23 patients per group were required to establish variance tests with *α* = 0.05 and *β* = 0.1. Ultimately, data from 56 patients were calculated to account for the anticipated 20% dropout rate.

SPSS software from Windows 19.0 (SPSS, Chicago, IL, USA) was used for statistical analysis. For continuous variables, the Kolmogorov-Smirnov test was used to assess the normality of the data. Continuous variables were summarized as mean ± standard deviation, median and interquartile ranges calculated, and analyzed using the Student’s *t* test or a Mann-Whitney *U* test as appropriate. For categorical variables, data of the two study groups were summarized as a frequency, *n* (%), and the Pearson *c*^2^ test or Fisher exact test was performed as appropriate. *P* < 0.05 was considered statistically significant for all results.

## Results

The flow chart of this study is presented in Fig. 1. Sixty-five patients scheduled for RAPN were screened for eligibility, and 9 patients were excluded due to not meeting inclusion criteria or declining to participate. A total of 56 patients were randomized into two equally sized groups (*n* = 28, for each group) and were included in the final analysis. The results showed a significant reduction of remifentanil consumption in the QLB group compared to usage in the control group (1129.3±310.22 μg vs 1778.43±668.74 μg, *P< 0.001*). The two groups were similar regarding other demographic and anesthesia- and surgery-related characteristics (Table 1).

**Table 1:** Demographic and anesthesia- and surgery-related characteristics

	Control group (*n=28*)	QLB group (*n=28*)	*P* value
Age (years)	48.25±11.30	47.07±12.05	0.707
Sex (male/female)			0.284
Male	13 (46.43)	17 (60.71)	
Female	15 (53.57)	11 (39.29)	
BMI (kg/m^2^)	23.61±2.51	23.27±3.14	0.661
ASA (I/II/III)			0.540
I	9 (32.14)	11 (39.29)	
II	15 (53.57)	14 (50.00)	
III	4 (14.29)	3 (10.71)	
Surgical site (left/right)			0.593
Left	13 (46.43)	15 (53.57)	
Right	15 (53.57)	13 (46.43)	
Surgical duration (min)	187.68±49.08	187.75±53.57	0.996
Propofol consumption (mg)	1412.68±449.30	1366.25±464.85	0.705
Remifentanil consumption (μg)	1778.43±668.74	1129.39±310.22*	< 0.001
Length of PACU stay (min)	50.61±16.26	47.75±17.23	0.526
Hospital stay (days)	6.32±2.06	5.82±1.87	0.345

Data are presented as mean ± standard deviation or number (%)

*ASA* American Society of Anesthesiologists physical status, *BMI* body mass index, *PACU* post-anesthesia care unit

*Significant (*P* < 0.05) difference between groups

VAS assessments (pain scores at rest and during movement) at all predetermined intervals are shown in Table 2. Pain scores during movement at 6 h post operation (primary outcome) were statistically significantly lower in the QLB group than scores in the control group (3.4±1.1 vs 4.4±1.1, *P* < 0.001). Meanwhile, VAS scores during movement at all times except 4 h were statistically lower in the QLB group (all *P* < 0.05). VAS scores at rest for most of the predetermined intervals, including at the PACU and at 4, 12, and 24 h, were statistically significantly lower in the QLB group than scores in the control group (all *P* < 0.05). In addition, during the first 6 h after surgery (the period of morphine titration and PCA use), patients in the QLB group used significantly less morphine than those in the control group (all *P* < 0.05), but not at 12, 24, and 48 h (Table 3).

**Table 2:** Visual analog scale pain scores at rest and movement

Measurement time	Control group (*n=28*)	QLB group (*n=28*)	*P* value
Rest			
4 h	1.93±0.86	1.43±1.03*	0.022
6 h	3.04±0.88	1.82±0.77*	< 0.001
12 h	2.64±1.06	1.50±0.69*	0.001
24 h	2.04±0.74	1.32±0.82*	0.008
48 h	1.46±0.64	0.96±0.69	0.076
Movement			
4 h	2.61±0.88	2.07±0.72	0.075
6 h	4.4±1.1	3.4±1.1*	< 0.001
12 h	3.46±1.10	2.32±0.67*	0.001
24 h	3.29±0.85	2.57±0.88*	0.002
48 h	2.61±0.63	2.25±0.65*	0.013

Data are presented as mean ± standard deviation

*Significant (*P* < 0.05) difference between groups

**Table 3:** Cumulative morphine consumption

Measurement time	Control group (*n=28*)	QLB group (*n=28*)	*P* value
Morphine titration consumption (mg)	2.00 (1.00, 4.00)	2.00 (0.00, 2.00)*	0.037
Morphine PCA use (mg)			
Titration			
4 h	1.00 (0.50, 2.00)	0.00 (0.00, 1.00)*	0.002
6 h	3.00 (2.50, 4.00)	2.00 (1.00, 2.50)*	< 0.001
12 h	8.00 (6.00, 10.50)	7.00 (6.00, 8.00)	0.089
24 h	13.00 (10.50, 16.00)	13.00 (11.50, 14.00)	0.718
48 h	17.00 (14.00, 19.00)	16.50 (15.00, 19.50)	0.617

Data are presented as median (IQR)

*IQR* interquartile range, *PCA* patient controlled analgesia

*Significant (*P < 0.05*) difference between groups

There was a significant difference in incidences of nausea and vomiting between the two groups (all *P* < 0.05), though other morphine-related side effects were similar across the groups (all *P* > 0.05) (Table 4). However, the mean (SD) QoR-15 quality of recovery scores were 75.4 (4.6) and 83.5 (4.5) for patients receiving QLB and no block, respectively (*P* < 0.001) (Table 5). There were significant differences in five sub-items of QoR-15, namely: “Have had good sleep,” “Able to look after personal hygiene, urination, and defecation unaided,” “Moderate pain,” “Severe pain,” and “Nausea or vomiting” (all *P*< 0.05).

**Table 4:** Morphine-related side effects

	Control group (*n=28*)	QLB group (*n=28*)	*P* value
Hyoxemia *n* (%)	2 (7.1%)	0	0.245
Sedation *n* (%)	9 (32.1%)	4 (14.3%)	0.114
Dizziness *n* (%)	10 (35.7%)	6 (21.4%)	0.237
Nausea *n* (%)	12 (42.9%)	5 (17.9%)*	0.042
Vomiting *n* (%)	8 (28.6%)	2 (7.1%)*	0.036
Pruritus *n* (%)	6 (21.4%)	4 (14.3%)	0.485

Data are presented as number (%)

*Significant (*P* < 0.05) difference between groups

**Table 5:** QoR-15 items at day 1 postoperatively

	Control group (*n=28*)	QLB group (*n=28*)	*P* value
Able to breathe easily	6.0 (1.4)	6.3 (1.3)	0.559
Able to enjoy food	5.4 (1.6)	5.5 (1.3)	0.788
Feeling energized	5.2 (1.4)	5.2 (1.5)	1.000
Have had a good sleep	3.4 (1.1)	5.1 (1.5)*	< 0.001
Able to look after personal hygiene, urination and defecation unaided	2.2 (1.0)	3.0 (0.8)*	0.002
Able to communicate with family and friends	7.0 (1.5)	7.0 (1.3)	1.000
Getting support from hospital, doctors and nurses	6.5 (1.0)	6.9 (1.1)	0.190
Able to return to work or usual home activities	1.4 (1.0)	1.7 (0.9)	0.275
Feeling comfortable and in control	7.1 (1.0)	7.0 (1.0)	0.697
Having a feeling of general well-being	6.9 (0.9)	7.0 (1.4)	0.815
Moderate pain	3.3 (1.3)	4.8 (1.0)*	< 0.001
Severe pain	5.0 (1.0)	6.4 (1.2)*	< 0.001
Nausea or vomiting	3.5 (1.8)	5.1 (1.9)*	0.003
Feeling worried or anxious	6.0 (1.7)	6.2 (1.6)	0.744
Feeling sad or depressed	6.2 (1.3)	6.5 (1.4)	0.438
Total score	75.4 (4.6)	83.5 (4.5)*	< 0.001

Data are presented as mean±standard deviation

*QoR-15* 15-item quality of recovery core

*Significant (*P* < 0.05) difference between groups

## Discussion

The current study was designed to evaluate the perioperative analgesic efficacy of a preoperative ultrasound-guided single-injection QLB in patients undergoing RAPN under general anesthesia. The main finding of this study was that a QLB provided superior analgesia at the early postoperative stage resulting in lower pain scores, less opioid consumption, and fewer adverse reactions. We found that the preventive implementation of a QLB reduced pain scores at rest during any time point up to 24 h. VAS scores during movement were significantly lower in the QLB group at all observed intervals except at 4 h postoperatively. Similarly, the consumption of intraoperative remifentanil or postoperative morphine during the first 6 h, but not thereafter, was significantly lower in the QLB group than in the control group. Hence, the QLB may be a useful analgesic method along with typical patient-controlled analgesia for RAPN.

The ultrasound-guided QLB technique was first described by Blanco [11, 12], and the benefits of QLB for postoperative pain relief and an opioid-sparing effect have been reported by several randomized controlled trials and case reports [2, 5, 6, 8–10, 19–30]. All approaches have proven the synergistic efficacy for multimodal analgesia, especially for QLB2 or QLB3 after laparoscopic surgery, cesarean section, and total hip arthroplasty. This is the first randomized, double-blinded, controlled trial study that has compared the preventive implementation of QLB2 to a standard perioperative analgesic regimen when applied in RAPN. For most studies, the QLB2 (posterior approach) was chosen as its targeted injection was more superficial, focused, and easily positioned. Our findings echo most previous trials. Irwin et al. [10] investigated the posterior approach for postoperative pain relief after cesarean section and showed a reduction in median (IQR [range]) VAS pain scores at 6 h postoperatively. However, opioid consumption was similar in both groups during the first 24 h after surgery. Kukreja et al. [29] also demonstrated the benefits of an opioid-sparing analgesic effect of the anterior QLB in total hip arthroplasty. The true mechanism of a QLB is not completely known. One of the critical diffusion mechanisms of both anterior and posterior approaches is the spread of LA to the paravertebral space region to achieve effective analgesia in the desired dermatomes. However, the postulation of LA consistently tracking into the anterolateral penetration of quadratus lumborum and anterior thoracolumbar fascia has been called into question [31, 32]. It remains to be seen whether a different approach, such as intramuscular or lateral, would provide superior or longer lasting analgesia.

Multimodal analgesia has become standard for postoperative pain management in our surgical center. It consists of preoperative prophylactic analgesia, combined different analgesic medications, local infiltration anesthesia, and patient-controlled intravenous analgesia. We therefore did not directly compare the use of QLB with a sham block group (same volume of saline), epidural analgesia, or other approaches, as we felt that the aforementioned analgetic scheme has remarkable efficacy in pain relief, especially for patients who are involved in enhanced recovery programs. Therefore, this study focused on whether QLB enables an increased analgesic effect and the role it plays in typical multimodal analgesia [33, 34]. A recent study by Aditianingsih et al. [2] compared the anterior approach with epidural analgesia in patients undergoing laparoscopic donor nephrectomy. They demonstrated that morphine consumption and pain scores at 24 h after surgery were comparable. A propensity score-matching analysis has illustrated that postoperative pain was not significantly different between the different operation modes (RAPN vs laparoscopic partial nephrectomy) [35].

Postoperative pain management has always been a core value to enhanced recovery after surgery. We took this opportunity to use the QoR-15 developed by Stark et al. [36] as a secondary outcome measure. As in previous studies, the pain scores were highest within 24 h after surgery and must be noted because severe pain is associated with a series of adverse reactions after surgery and anesthesia. However, the QoR-15 also provides a quantitative measure of comfort, emotional wellbeing, and physical functioning and can assess functional recovery rather than pain. In this study, there were significant statistical differences in five sub-items (have had good sleep; able to look after personal hygiene, urination, and defecation unaided; moderate pain, severe pain, and nausea or vomiting) and total scores between the groups at 24 h after surgery. Therefore, it could be that single-injection QLB may not merely relieve acute pain; it may also serve a supportive role for improving overall health status at the early stages after surgery.

## Limitations

There are some limitations to our study. Firstly, VAS scores during movement at 6 h postoperatively were chosen as the primary outcome measure. VAS scores in a pilot study and the present study were tested to conform to a normal distribution. A Student’s *t* test was used to analyze pain scores at each predetermined interval. Secondly, we did not check sensory dermatomal levels, assess visceral pain, record lower extremity weakness, or accurately calculate the duration of the QLB to explain the characteristics of the sensory blockade. The main purpose of this study was to compare pain scores and opioid consumption between the two groups. Thirdly, some studies have identified a phenomenon referred to as “rebound pain” after a single-shot peripheral nerve block, which is defined as very severe pain when the peripheral nerve block wears off. It is not a rare problem in clinical practice and could affect up to 40% of patients undergoing orthopedic procedures [37], but its pathophysiological mechanisms remain unknown. The QoR-15 collected all related data at 24 h post-surgery, such as “Have had good sleep” and “Severe pain,” which might provide a bit of tracing data in regard to rebound pain. However, we did not observe that severe pain interfered with sleep or complaints of severe discomfort when the QLB wore off. In the future, randomized controlled trials may be needed to include all the aforementioned indicators which may impact a pain management program and patient satisfaction.

## Conclusions

In conclusion, a posterior QLB enabled adequate postoperative analgesia for patients undergoing RAPN, as it reduced pain scores and had opioid-sparing effects postoperatively. The preoperative implementation of a single-shot QLB is an important component of multimodal analgesia and may be helpful to enhance functional recovery. Further studies are warranted to determine the best approach as well as the optimal dose and volume of LA or adjuncts required for a more effective and lasting QLB for patients receiving RAPN.

## Data Availability

The datasets used and/or analyzed during the current study are available from the corresponding author on reasonable request.

## Abbreviations

QLB: Quadratus lumborum block
LA: Local anesthetic
RAPN: Robot-assisted partial nephrectomy
ASA: American Society of Anesthesiologist
BMI: Body mass index
VAS: Visual analog scale
PCA: Patient controlled analgesia
BIS: Bispectral index
TCI: Target-controlled infusion
TOF: Train-of-four
PACU: Post-anesthesia care unit
QoR-15: 15-item quality of recovery score
IQR: Interquartile range
